# Valorization of Pretreated *Salvinia molesta* Biomass for Ciprofloxacin Biosorption: Kinetic Performance, pH‐Dependent Mechanisms, and Circular Economy Implications

**DOI:** 10.1002/wer.70304

**Published:** 2026-02-15

**Authors:** Leticia Yoshie Kochi, Raul Victor Santana Rios, Raizza Zorman Marques, Lia Sumie Nakao, Marcelo Pedrosa Gomes

**Affiliations:** ^1^ Laboratório de Fisiologia de Plantas Sob Estresse, Departamento de Botânica, Setor de Ciências Biológicas Universidade Federal do Paraná Curitiba Paraná Brazil; ^2^ Departamento de Patologia Básica, Setor de Ciências Biológicas Universidade Federal do Paraná Curitiba Paraná Brazil

**Keywords:** aquatic macrophytes, ciprofloxacin removal, sustainable water treatment, waste biomass valorization

## Abstract

The valorization of pretreated waste *Salvinia molesta* biomass represents a sustainable and circular strategy to address both water contamination and biomass disposal. This study investigated the biosorption performance of pretreated and powdered *S. molesta* biomass in controlled aqueous solutions of ciprofloxacin (CIP), a widely detected fluoroquinolone antibiotic, under environmentally relevant conditions. The biomass was characterized by a high cell wall fraction (~61%) and moderate protein and polyphenol content, offering a multifunctional surface for biosorption. Batch experiments were conducted to evaluate the effects of pH (4–8) and contact time (up to 60 min) on CIP removal (initial concentration = 1.5 μg/L). The maximum biosorption efficiency (~95%) occurred at pH 6, which aligned with the biomass's point of zero charge (${\text{pH}}_{p}\text{zc}$ = 6.2) and the CIP zwitterionic speciation. Biosorption was rapid in the first 30 min, although equilibrium was rapidly reached within 30 min, consistent with the classical biosorption behavior of dead biomass at low concentrations. The kinetic model followed a pseudo‐second‐order trend, which was interpreted empirically rather than mechanistically, reflecting surface‐controlled adsorption dynamics. Pearson correlations revealed that the protein and polyphenol contents were positively associated (*r* > 0.85) with biosorption at pH 6–7, highlighting a multi‐mechanistic interaction involving electrostatic, hydrogen bonding, and π–π interactions. These findings suggest that *S. molesta* is a naturally abundant, low‐cost biosorbent suitable for decentralized water remediation, particularly in small‐scale or proof‐of‐concept systems using model aqueous matrices, with potential applications in passive treatment units and community‐based sanitation systems. Future studies should evaluate isotherm behavior, thermodynamic parameters, and the regeneration potential of the biosorbent to determine scalability under real wastewater conditions.

## Graphical abstract

Powdered *Salvinia molesta* biomass efficiently removed ciprofloxacin under environmentally relevant conditions, with optimal sorption at a near‐neutral pH. This study highlights invasive plant biomass valorization as a low‐cost circular solution for decentralized water treatment.

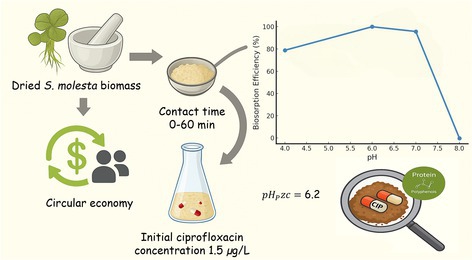

## Summary

- Powdered pretreated waste biomass of *Salvinia molesta* efficiently removed ciprofloxacin from controlled aqueous solutions.
- Maximum removal occurred at pH 6–7, matching surface charge and drug speciation.
- Pseudo‐second‐order kinetics indicated surface‐controlled interactions, not chemisorption.
- Equilibrium reached within 30 min under environmentally realistic concentrations.
- Valorization of waste macrophyte biomass supports circular economy water treatment.

## 1 Introduction

The widespread presence of pharmaceutical contaminants in aquatic ecosystems is a pressing environmental and public health concern. Among these, antibiotics such as ciprofloxacin (CIP), a broad‐spectrum fluoroquinolone, are frequently detected in surface water and wastewater treatment plant (WWTP) effluents at concentrations ranging from a few ng/L to 76 μg/L (Marques, da Silva Nogueira, et al. 2024; Marques, Oliveira, et al. 2024; Nogueira et al. 2025; Le et al. 2016). Their persistence and incomplete removal by conventional treatment technologies underscore the need for efficient and low‐cost alternatives to mitigate pharmaceutical pollution (Marques, da Silva Nogueira, et al. 2024; Marques, Oliveira, et al. 2024).

Biosorption has emerged as a promising approach for removing micropollutants from aqueous media. Biosorption involves passive interactions between pollutants and biological materials rich in functional groups capable of binding diverse chemical species (Deyris et al. 2023). Although various biosorbents, including algae, fungal biomass, and agricultural residues, have been studied, the vast, underutilized biomass of aquatic macrophytes remains untapped (Velkova et al. 2025; Karnwal 2024). Aquatic macrophytes are often regarded as nuisances or ecological threats because of their rapid growth and proliferation in nutrient‐rich environments. Species such as *Salvinia molesta* are commonly removed mechanically or chemically to control eutrophication in lakes and reservoirs (Schneider et al. 2024). However, these control efforts frequently overlook the ecological and material potential of these plants (Sayanthan et al. 2024). In their living form, macrophytes have demonstrated the capacity to take up antibiotics, including CIP, primarily through passive uptake mechanisms associated with a transpiration‐driven flow (Marques, Oliveira, et al. 2024; Gomes et al. 2018; Maranho and Gomes 2024). This raises a critical question: If living plants can passively or actively accumulate such contaminants through uptake and partial metabolism, can their dead biomass be repurposed as an efficient biosorbent?

The valorization of pretreated or waste macrophyte biomass represents a sustainable and circular strategy to address both aquatic contamination and the challenge of biomass overaccumulation. Previous studies have demonstrated the biosorption capacity of *S. molesta* for heavy metals, dyes, and pollutants (Sharma et al. 2023; Rachmadiarti et al. 2022; Dolui et al. 2025). However, few studies have explored its performance in the removal of antibiotics at environmentally realistic trace concentrations. Transforming invasive aquatic plants into biosorptive media not only provides a low‐cost and renewable resource for water treatment but also opens avenues for local income generation through harvesting, processing, and reuse (Zoppi et al. 2024). Furthermore, this approach supports environmental education by reinforcing the ecological roles of macrophytes and highlighting their potential in biotechnological applications. Despite the ecological burden imposed by invasive species such as *S. molesta* , its prolific growth can be regarded as a technological opportunity. Unlike most studies relying on chemically modified or activated biosorbents, our approach emphasizes the use and alkaline pretreatment of dried and minimally pretreated *S. molesta* biomass. This highlights a paradigm shift from high‐cost activation to the ecological valorization of naturally abundant biomass, particularly in the context of decentralized and low‐tech water treatment solutions. The use of dried and minimally pretreated *S. molesta* as a biosorbent, as proposed in this study, offers an environmentally practical and socially inclusive alternative for the removal of contaminants.

Therefore, we investigated the biosorption capacity of pretreated and powdered *S. molesta* biomass for CIP, a widely detected fluoroquinolone antibiotic, under pH conditions representative of both surface water and treated effluents in controlled aqueous solutions. We examined environmentally realistic concentrations (1.5 μg L^−1^), linking the chemical composition of the biomass (protein and polyphenol content) with biosorption performance. Kinetic models were applied to characterize the adsorption dynamics, and the influence of surface charge behavior ($pH_{P}\mathrm{zc}$) was assessed under different conditions. This study provides novel insights into the valorization of invasive macrophyte biomass for antibiotic removal, reinforcing the potential of waste plant material as a circular economy tool for decentralized water remediation.

## 2 Material and Methods

### 2.1 Biomass Collection and Preparation

Specimens of *S. molesta* were collected from an artificial lentic water body located on the Jardim Botânico campus of the Federal University of Paraná (UFPR), Curitiba, Paraná, Brazil (25°26′52.7″ S, 49°14′13.3″ W). The site is characterized by nutrient‐rich and eutrophic conditions and is used for teaching and research. After manual harvesting, the plants were transported to the laboratory in sealed polyethylene containers and washed thoroughly with distilled water to remove epiphytes, sediments, and other associated particulate matter. The clean biomass was then oven‐dried at 60°C under forced air circulation until a constant weight was achieved (approximately 72 h). The dried material was ground using an electric grinder (Cadence Di Grano MDR302‐127), thereby enabling the production of smaller and more uniform particles. The ground biomass was then sieved using a 0.5‐mm mesh, and the fraction retained between the two mesh sizes (0.5–1.18 mm) was selected for use in the biosorption experiments (Saralegui et al. 2021). To remove pigments and soluble organic compounds, an alkaline pretreatment with 0.1 mol L^−1^ NaOH for 2 h (120 rpm) was performed, followed by successive washes with distilled water, 70% ethanol, and 80% acetone. The biomass was then dried at 60°C for 72 h until all moisture was removed. This pretreatment constitutes a chemical modification of the biomass and was chosen for its low reagent demand, recoverability, and established use in green‐chemistry biomass valorization, ensuring cost‐effective and environmentally compatible preparation. The final pH of each treatment was measured at the end of incubation, and values remained within 0.2 pH units of initial conditions.

### 2.2 Biosorption Assays

**FIGURE 1:**
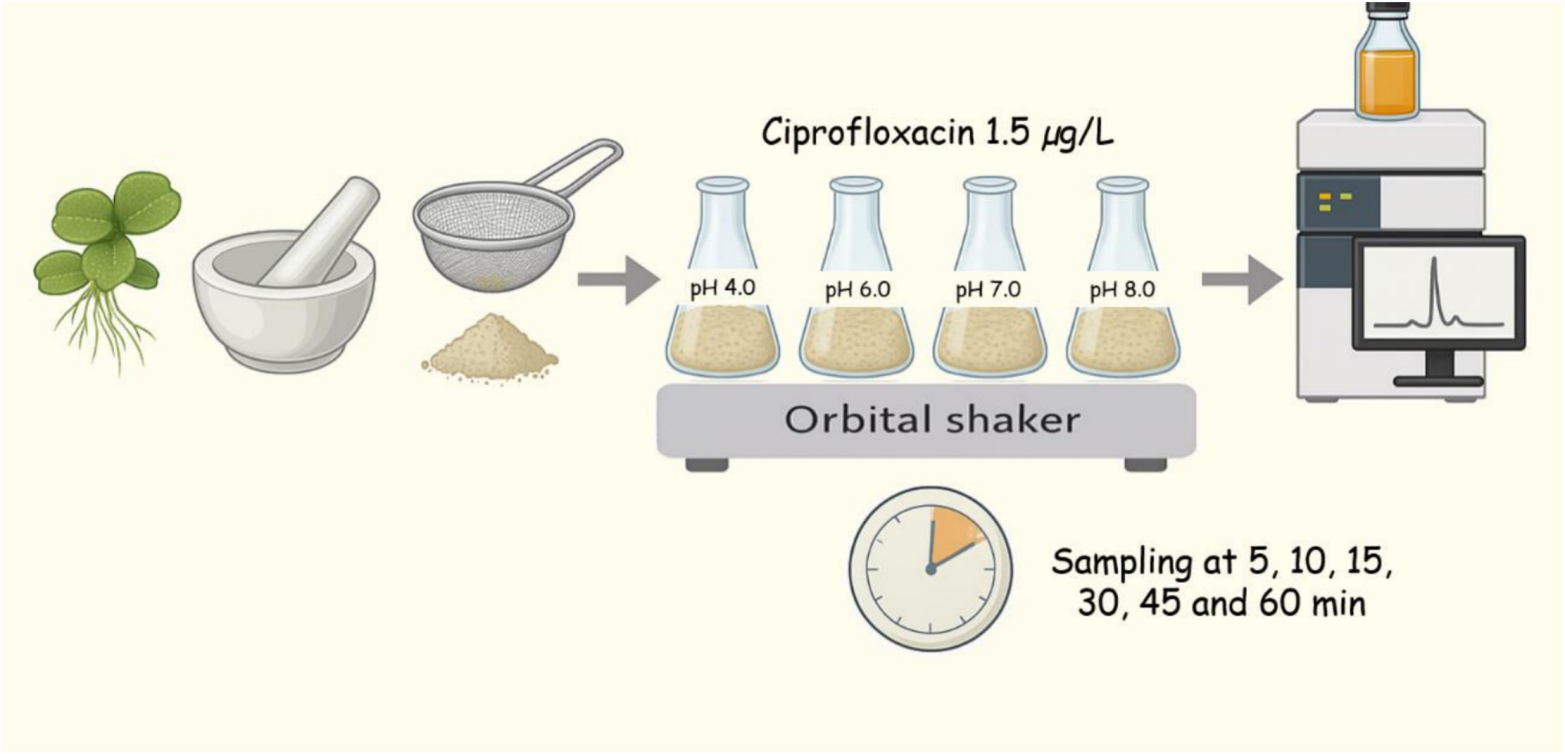
Schematic representation of the experimental procedure used to evaluate ciprofloxacin biosorption by dried and ground *Salvinia molesta* biomass. The process involved biomass collection, drying, grinding, incubation with ciprofloxacin solution (up to 60 min), and quantification of the remaining concentration to estimate biosorption parameters.

CIP was selected as the target contaminant because it is frequently detected in effluents and surface water owing to its broad‐spectrum activity and limited biodegradability (Marques et al. 2021). Analytical‐grade ciprofloxacin hydrochloride (≥ 98% purity, Sigma‐Aldrich, USA) was used to prepare an aqueous solution (1.5 μg L^−1^) in ultrapure water, representing environmentally realistic concentrations. Biosorption assays were conducted in 100‐mL Erlenmeyer flasks containing 50 mL of CIP solution and 0.25 g of pretreated *S. molesta* biomass. A control group without biomass was used to assess the abiotic losses. Assays were conducted at 20°C using an orbital shaker (KLA‐210, VDRL/Kline type) set at 120 rpm for 60 min (Khokhar et al. 2019). Four pH values—4.0, 6.0, 7.0, and 8.0—were tested and adjusted using 0.1 mol L^−1^ HCl or NaOH to represent typical conditions of domestic and industrial effluents (Marques, Oliveira, et al. 2024). At 0, 5, 10, 15, 30, 45, and 60 min, 2 mL aliquots were sampled, filtered through 0.45‐μm nylon membranes, and immediately analyzed. All treatments were performed in triplicates. The full experimental workflow is shown in Figure 1.

### 2.3 Analytical Determination of CIP

The residual CIP concentrations were quantified using high‐performance liquid chromatography coupled with ultraviolet/visible (UV/VIS) detection (Kochi et al. 2023). Detection was performed at 278 nm using a Shimadzu LC‐20AD with a C18 column (150 × 4.6 mm, 5 μm). Linearity (*R* ^2^ > 0.998), detection (0.005 μg L^−1^) and quantification (0.010 μg L^−1^) limits, recovery > 95%, and RSD < 5% confirmed the method's reliability. The system consisted of a Shimadzu LC‐20AD unit equipped with a C18 reverse‐phase column (150 × 4.6 mm, 5 μm). The mobile phase consisted of 0.1% formic acid in ultrapure water and acetonitrile (80:20, v/v) at a flow rate of 1.0 mL/min. The injection volume was 20 μL. Detection was performed at 278 nm using a UV/VIS detector. For complementary fluorescence detection, the excitation and emission wavelengths were set at 275 and 450 nm, respectively. Calibration curves were constructed in the 0.01–10.0 μg/L range (*R* ^2^ > 0.998). The detection and quantification limits (MDL and MQL, respectively) were 0.005 and 0.010 μg/L, respectively. The specificity was confirmed by matching the retention times and UV spectra with authentic CIP standards. Linearity was maintained across the entire calibration range, with *R* ^2^ consistently above 0.998. The robustness of the method was verified by assessing the retention time stability and response under variations in the mobile phase composition (±5%) and flow rate (±0.1 mL/min), with a relative standard deviation (RSD) of < 5%. Matrix recovery, tested using CIP‐spiked biomass extracts, was > 95%, confirming negligible matrix interference (Figure S1). The analytical method was validated in terms of linearity, sensitivity, precision, recovery, and matrix effects, confirming its suitability for low‐level CIP quantification in aqueous matrices.

### 2.4 Calculation of Biosorption Capacity

The amount of sorbed CIP at time *t* (qt, μg/g) was calculated at each time point (Table S2) using the mass balance equation (Equation 1). The equilibrium capacity (qe) was defined at 60 min, corresponding to the kinetic equilibrium rather than an isotherm‐derived qmax, because only a single initial concentration was tested. Abiotic losses were subtracted from the final values.

(1) $$\mathrm{qt}=\frac{\left(C_{0}-C_{t}\right)\times V}{m}$$

where

- *C₀* = initial CIP concentration (μg/L)
- *C*_*t*_ = CIP concentration at time *t* (μg/L)
- *V* = volume of solution (L)
- *m* = biomass mass (g)

The equilibrium value (qe) was defined at 60 min, and abiotic losses determined in the control group were subtracted from the final values to correct for nonbiological removal.

### 2.5 Kinetic Modeling

To describe the biosorption kinetics, the experimental data were fitted to pseudo‐first‐ and pseudo‐second‐order models using nonlinear regression:

Pseudo‐first‐order model:

(2) $$\mathrm{qt}=\mathrm{qe}\times \left(1-e^{-k_{1}t}\right)$$

Pseudo‐second‐order model:

(3) $$\mathrm{qt}=\frac{{\mathrm{qe}}^{2}\times k_{2}\times t}{1+\mathrm{qe}\times k_{2}\times t}$$

where qe is the maximum biosorption capacity (μg/g), *k*_*1*_ and *k*_*2*_ are kinetic constants (min^−1^), and *t* is the time (min). These kinetic models were selected because of their widespread use in biosorption studies, which facilitates comparison with previous research. Although the pseudo‐second‐order model provided the best fit (*R* ^2^ > 0.98), it was interpreted empirically, describing adsorption dynamics under low‐concentration conditions, rather than as definitive evidence of chemisorption. This distinction was made in response to previous reports (Mohamed Nasser et al. 2024; No Title, n.d.), indicating that a good pseudo‐second‐order fit alone does not prove that chemical bonding is the rate‐limiting step. Therefore, our interpretation emphasizes that the model represents the kinetics of sorption under low‐concentration conditions, rather than the mechanistic nature of the process. The best‐fit models were selected based on the coefficient of determination (*R* ^2^) and residual error analyses.

### 2.6 Biomass Chemical Characterization

The chemical composition of *S. molesta* biomass was evaluated to determine the factors influencing biosorption capacity. The analyses were conducted in quintuplicate (*n* = 5). Total nitrogen was determined using the Kjeldahl method, and the crude protein content was calculated using a conversion factor of 6.25 (Boyd 1968). The ash content was quantified by incineration at 550°C for 4 h. Soluble carbohydrates were quantified using the phenol–sulfuric acid method (DuBois et al. 1956). The cell wall fraction was assessed using neutral detergent extraction (Van Soest 1990). Polyphenols were extracted using methanol and quantified spectrophotometrically using the Folin–Ciocalteu reagent (King and Heath 1967).

### 2.7 Statistical Analysis

All experimental results are expressed as mean ± standard deviation (SD) for *n* = 5. One‐way analysis of variance (ANOVA) followed by Tukey's honest significant difference (HSD) test (*p* < 0.05) was used to evaluate the statistically significant differences among the pH treatments. Pearson's correlation coefficients were calculated to assess the relationship between the chemical composition of biomass and the biosorption efficiency across pH levels. All regression analyses, including nonlinear kinetic modeling, correlation matrices, and data visualization (heatmaps and fitted curves), were performed using RStudio (v4.3.0) and GraphPad Prism 10.

## 3 Results and Discussion

### 3.1 Biomass Characterization and Biosorptive Potential

The dried pretreated biomass of *S. molesta* used in this study exhibited diverse biochemical compositions (Table 1). The cell wall fraction was particularly high (61.13 ± 1.12%), indicating a substantial amount of lignocellulosic material in the biomass. The crude protein content reached 17.85 ± 0.64%, and polyphenols totaled 0.83 ± 0.04 mg GAE/g. Although these values are lower than those typically found in protein‐rich aquatic microalgae (e.g., *Chlorella* spp.), they compare favorably with other macrophyte‐based biosorbents previously reported for metal and dye removal (Haroon 2020). The lipid (~5%) and ash (~16%) contents were also consistent with the composition of structural aquatic plant tissues (Haroon 2020).

**TABLE 1:** Chemical composition of *Salvinia molesta* biomass used in the biosorption assays (*n* = 5).

Parameter	Value (mean ± SD)
Crude protein (%)	17.85 ± 0.64
Ash content (%)	15.86 ± 0.42
Total nitrogen (%)	2.60 ± 0.09
Cell wall fraction (%)	61.13 ± 1.12
Polyphenols (UDO g^−1^)	0.83 ± 0.04
Soluble carbohydrates (mg/L)	16.47 ± 0.93
Lipids (%)	4.98 ± 0.27

Compared to other aquatic species, *S. molesta* has a moderate protein content but is distinguished by its elevated cell wall fraction. Aquatic macrophytes such as *Lemna minor* and *L. gibba* often have protein contents between 20% and 45% (Iatrou et al. 2019; Mirzaeva et al. 2020), whereas microalgae such as *Chlorella* and *Spirulina* exceed 30% (Song et al. 2025). Although *S. molesta* has lower protein levels, the structural richness of its cellulose and lignin matrix, as evidenced by the large cell wall fraction, is likely the principal contributor to biosorption, especially when coupled with detectable amounts of aromatic polyphenols, such as quercetin, gallic acid, and caffeic acid, previously identified in *S. molesta* extracts (Song et al. 2025; Kumar et al. 2022). The presence of protein‐derived functional groups (−NH_2_, −COOH), polysaccharide hydroxyls (−OH), and aromatic phenolic rings offers a composite surface chemistry capable of engaging CIP via hydrogen bonding, electrostatic attraction, and π–π interactions (Feizi and Fatehi 2021). Pearson correlation analysis indicated strong positive relationships for protein (r_ph6_ = 0.86; r_ph7_ = 0.89) and polyphenols (r_ph6_ = 0.88; r_ph7_ = 0.86) between the removal efficiency at pH 6–7 and these two components (*p* < 0.05), reinforcing a multi‐mechanistic sorption process. These findings parallel previous reports of fluoroquinolone adsorption onto biosorbents enriched with amine and phenolic moieties (Duan et al. 2020; Tolić et al. 2021; Jiang et al. 2021).

### 3.2 pH‐Dependent Biosorption Efficiency

**FIGURE 2:**
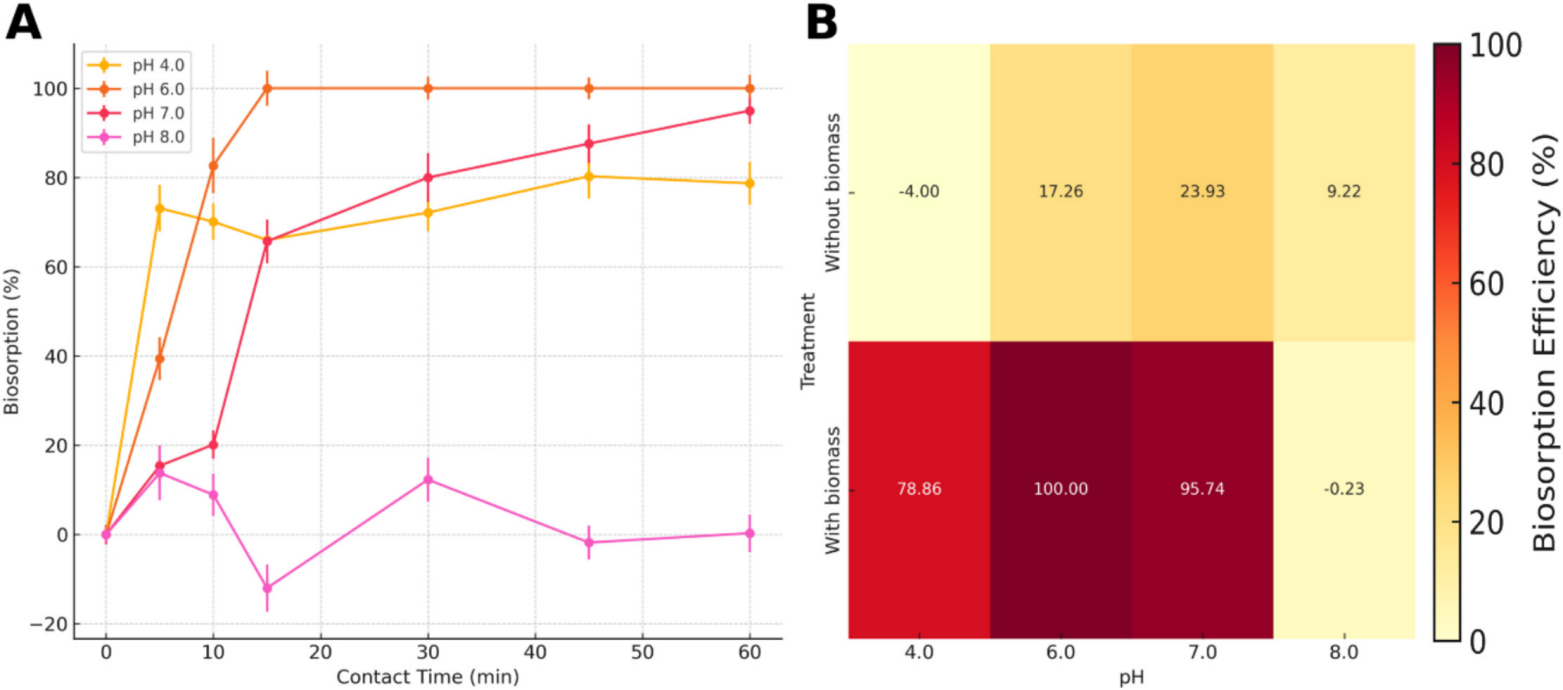
(A) Biosorption efficiency (%) of ciprofloxacin on *Salvinia molesta* biomass over time under different pH conditions (4.0, 6.0, 7.0, and 8.0). Data are presented as mean ± standard deviation (*n* = 5). (B) Heatmap of ciprofloxacin removal efficiency (%) after 60 min of contact, showing biosorption performance across different pH values and treatments (with and without biomass). The values represent the means of five replicates.

Figure 2A,B and Table S1 show the influence of pH and contact time on CIP removal. The highest biosorption efficiency (approximately 100%) was observed at pH 6, which closely matched the point of zero charge (${\text{pH}}_{p}\text{zc}=6.2$) determined for the *S. molesta* biomass (Figure S2). This condition favors the zwitterionic form of CIP and enables optimal electrostatic and hydrogen bonding interactions between the drug and biomass surface (Atugoda et al. 2021; Fu et al. 2021). At pH 4, where CIP is predominantly cationic, the biosorption efficiency dropped to approximately 79%, likely because of the electrostatic repulsion between the positively charged drug and positively charged biomass surface. Conversely, at pH 8, where CIP is predominantly anionic, the biosorption efficiency is negligible (~0%), indicating a complete loss of favorable interactions, such as hydrogen bonding and π–π stacking (Kini et al. 2025; Dou et al. 2022). The heatmap (Figure 2B) confirmed these trends, illustrating consistent performance across the five replicates and highlighting the importance of near‐neutral pH for maximizing biosorption. These findings emphasize that both environmental pH and the ionization state of the target compound play critical roles in determining biosorption success.

### 3.3 Kinetic Behavior and Model Fitting

**FIGURE 3:**
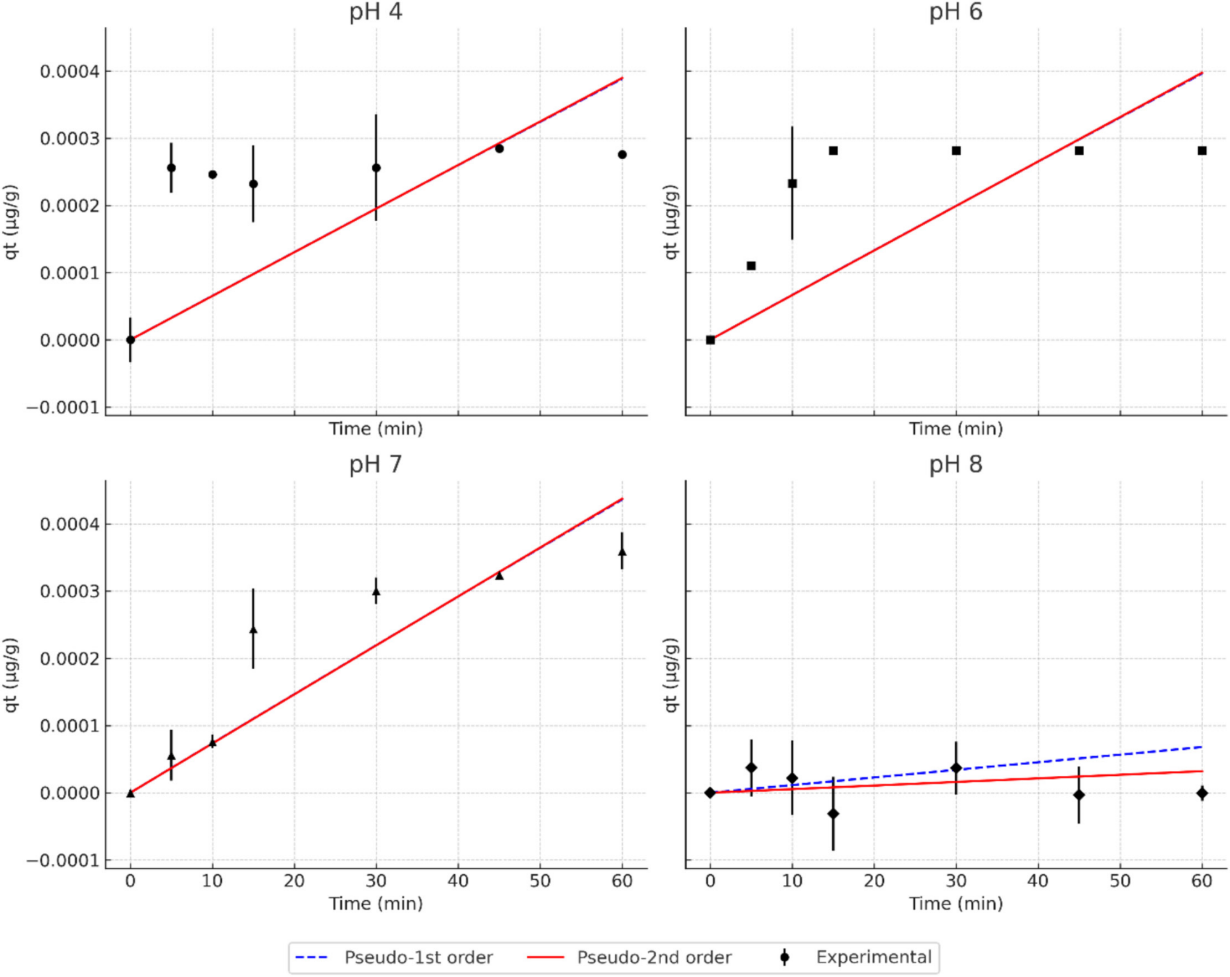
Ciprofloxacin biosorption kinetics (qt, μg/g) by *Salvinia molesta* biomass at different pH values (initial concentration = 1.5 μg/L; mass = 0.25 g; volume = 50 mL). Error bars represent the standard deviation (*n* = 3).

The biosorption kinetics of CIP at different pH values are presented in Figure 3. Across all treatments, qt values (uptake at time *t*) increased rapidly during the first 15–30 min, followed by a plateau phase, which is indicative of active site saturation. The uptake at time *t* (qt) mirrored these trends, with the highest values observed at pH 6 and 7. Model fitting showed that both pseudo‐first‐order and pseudo‐second‐order equations adequately described the data, with pseudo‐second‐order models generally yielding higher coefficients of determination (*R* ^2^ > 0.98; Table 2). Although the pseudo‐second‐order model provided the best statistical fit, this result was interpreted as an empirical correlation rather than definitive evidence of chemisorption. This clarification aligns with the literature, which indicates that a high *R* ^2^ value for pseudo‐second‐order kinetics does not prove that chemical bonding is the rate‐limiting step (Mohamed Nasser et al. 2024; No Title, n.d.). Therefore, the model was only used to describe the adsorption rate under low‐concentration conditions. The rapid uptake and early equilibrium (≈30 min) reflect the abundance of accessible binding sites on the pretreated biomass and the short diffusion path of the small‐molecule antibiotics. The limited variability after equilibrium explains the model sensitivity to early‐stage data. Future kinetic studies should test intraparticle diffusion and Elovich models to explore transport effects under environmentally realistic concentrations.

**TABLE 2:** Kinetic parameters for ciprofloxacin biosorption by *Salvinia molesta* biomass across pH values using pseudo‐first‐order and pseudo‐second‐order models.

pH	qe 1st (μg/g)	k1 (L/min)	*R* ^2^ (1st)	qe 2nd (μg/g)	k2 (g/μg·min)	*R* ^2^ (2nd)
4.0	0.106	0.053	0.888	0.149	0.471	0.996
6.0	0.169	0.043	0.932	0.164	0.120	0.990
7.0	0.169	0.043	0.932	0.164	0.120	0.988
8.0	0.12	0.066	0.964	0.119	0.609	0.982

### 3.4 Literature Comparison and Practical Implications

Compared to recent biosorption studies, *S. molesta* biomass demonstrated a notable performance in removing CIP at environmentally relevant concentrations. For instance, microalgae such as *Chlorella vulgaris* and *Synechocystis* sp. have shown high effectiveness in CIP removal, although typically at initial concentrations in the mg/L range under optimized conditions, and often involve extraction or chemical modification steps (Salah et al. 2024). Despite these differences, biosorption in these systems also followed pseudo‐second‐order kinetics and involved functional groups such as carboxyl, amine, hydroxyl, and carbonyl groups, which are known to enhance the binding efficiency (Salah et al. 2024; Li et al. 2018). Similarly, studies using aquatic plant–based materials, such as *Enteromorpha prolifera* , have demonstrated that the pseudo‐second‐order model best describes CIP uptake, with Freundlich isotherms indicating heterogeneous surface interactions (Fu et al. 2021; Li et al. 2018; Wu et al. 2015). The present study differs from previous studies in that it evaluated pretreated *S. molesta* biomass at environmentally realistic CIP concentrations (1.5 μg L^−1^) without chemical activation, demonstrating that mild pretreatment is sufficient for efficient antibiotic removal. This simplicity strengthens the circular economy viability for decentralized water treatment. Our previous research with live *S. molesta* exposed to 1.5 μg L^−1^ CIP for 7 days showed 97% removal without phytotoxicity (Kitamura et al. 2023). The comparable performance between live uptake and dead‐biomass biosorption suggests that the mechanism is primarily physicochemical rather than metabolic, reinforcing the suitability of pretreated waste biomass in engineered systems. Direct comparison with studies reporting qmax values should be interpreted cautiously because our q_e_ values were obtained from a single‐point kinetic assay rather than multi‐concentration equilibrium modeling. From a technological standpoint, *S. molesta* offers affordability, ease of handling, and scalability, which are advantages over synthetic sorbents that require activation or synthesis. Moreover, the alkaline pretreatment likely altered surface chemistry and may have contributed to the abundance of accessible binding sites, enhancing biosorption performance even at trace concentrations.

### 3.5 Broader Implications and Reusability Considerations

This study demonstrated that pretreated waste *S. molesta* biomass can serve as a low‐cost and environmentally feasible biosorbent for the removal of fluoroquinolones. *S. molesta* has also been reported to retain sulfonamides, azithromycin, and other antibiotics (Marques, Oliveira, et al. 2024; Kochi et al. 2023; Rocha et al. 2022), indicating broad applicability. The optimal conditions identified—pH 6–7 and ~30‐min contact—are compatible with typical surface water and effluent characteristics. Scaling this process requires addressing biomass sourcing, processing and post‐use management. Strategies for the regeneration, stabilization, and safe disposal of spent biomass should be developed to prevent desorption and secondary contamination. Potential disposal routes include composting, anaerobic digestion, and thermal treatment. Despite logistical considerations, the ecological and social trade‐offs remain favorable compared to those of synthetic sorbents. Moreover, the tangible nature of macrophyte biomass offers opportunities for community‐based remediation initiatives and environmental education, enhancing its value as both a biotechnological and a pedagogical resource.

### 3.6 Limitations of the Study and Recommendations for Future Work

Despite promising results, several limitations restrict their direct application. A key constraint lies in the use of a single initial concentration of CIP (1.5 μg/L), which, although environmentally relevant, prevents the construction of adsorption isotherms and the estimation of the maximum sorption capacity using models such as Langmuir or Freundlich. This limits our ability to predict the biosorbent behavior across different contamination scenarios. Additionally, the thermodynamic parameters of the biosorption process, such as enthalpy, entropy, and Gibbs free energy changes, which are fundamental for understanding the spontaneity and energetic nature of the interactions involved, were not explored. However, the potential for biomass regeneration and reuse has not been investigated. This is a critical omission because sorbent reusability directly affects the operational feasibility and cost‐effectiveness of practical applications.

Moreover, structural and surface characterization of the biomass was not performed. Techniques such as Fourier transform infrared spectroscopy (FTIR), scanning electron microscopy coupled with energy‐dispersive x‐ray spectroscopy (SEM‐EDX), and Brunauer–Emmett–Teller (BET) analysis can provide valuable insights into the functional groups involved in biosorption, morphological surface changes, and surface area dynamics before and after adsorption. Their absence limits our mechanistic understanding of the interaction of CIP with the biomass matrix. The experiments were conducted in ultrapure water under controlled conditions, which, although necessary for isolating treatment effects, do not represent the complexity of real aquatic systems. Wastewater contains a multitude of potentially interfering substances, including dissolved organic matter, competing ions, and other micropollutants. These factors can significantly influence biosorption efficiency and should be considered in future research.

The post‐use assessment of biosorbents is equally important in this regard. No tests have been conducted to evaluate the environmental fate, toxicity, or biodegradability of the spent biomass. Without this information, it is impossible to ensure that the treatment process does not merely shift the pollutant burden from water to solid waste. Understanding the risks and behaviors of saturated biomass is essential for safe disposal and possible valorization routes. Therefore, future research should aim to expand the experimental scope by testing multiple CIP concentrations, which would enable isotherm modeling and allow for a more accurate estimation of the maximum sorption capacity. It is also essential to evaluate the performance of biosorbents in real wastewater matrices containing competing ions, organic matter, and variable pH values to better reflect environmental complexity. Regeneration studies should be conducted to determine the number of feasible reuse cycles and to assess the long‐term operational stability of the biosorbent. In addition, applying analytical techniques such as FTIR, SEM‐EDX, and BET will help elucidate the structural and chemical changes in the biomass before and after biosorption. Environmental safety assessments of spent biomass, including ecotoxicity assays and degradation studies, are equally important to ensure that posttreatment handling does not pose any additional risks. These results should be interpreted as proof of concept obtained under controlled aqueous conditions and not as a complete mechanistic evaluation of biosorption. Future studies should incorporate multi‐concentration equilibrium modeling, thermodynamic parameters, regeneration cycles, and surface characterization to determine scalability under real wastewater conditions.

## 4 Conclusion

This study demonstrated that pretreated *S. molesta* biomass is an effective biosorbent for the removal of ciprofloxacin at environmentally relevant concentrations. Rapid uptake was observed within the first 30 min, with equilibrium achieved at 60 min, particularly at pH 6, which matched the surface charge neutralization point of the biomass. Positive correlations between protein and polyphenol content and biosorption performance highlight the relevance of biomass biochemical composition in determining antibiotic removal efficiency. The use of pretreated *S. molesta* biomass provides a sustainable alternative to conventional sorbents, offering low‐cost, abundant material for water treatment applications and potentially contributing to circular economy frameworks through biomass valorization. These findings illustrate the feasibility of transforming invasive macrophyte biomass into functional biosorbents under controlled aqueous conditions, providing a proof of concept for decentralized remediation strategies. However, because this study was based on a single initial concentration, q_e_ reflects equilibrium uptake rather than maximum capacity (qmax) derived from isotherm models, and further investigation is necessary to determine biosorption performance across a wider range of contaminant levels. Future work should incorporate multi‐concentration isotherm modeling, regeneration cycles, and structural characterization to assess scalability and mechanistic behavior under real wastewater conditions. Overall, the biosorption of ciprofloxacin by *S. molesta* biomass represents a promising and sustainable approach, reinforcing the potential of plant‐derived waste materials as low‐cost biosorbents for antibiotic removal.

## Author Contributions

L.Y.K. and M.P.G. conceived and designed the experiments. L.Y.K. and R.Z.M. performed sampling, experiments, and analyses. L.Y.K. and M.P.G. prepared the manuscript. L.S.N. provided technical support. M.P.G. received financial support for this study. All the authors have read and approved the manuscript.

## Funding

This work was supported by the Fundacion Araucaria (SAN2021251000002) and the Conselho Nacional de Desenvolvimento Científico e Tecnológico (302226/2022‐2).

## Conflicts of Interest

The authors declare no conflicts of interest.

## Supporting information


**Table S1:** Mean ± standard deviation of ciprofloxacin concentrations (μg/L) for each time point and pH condition, with and without biomass.
**Figure S1:** HPLC chromatograms and calibration curves were used for ciprofloxacin quantification, including the detection and quantification limits of the method.
**Table S2:** qt values (μg/g) were calculated across all time points and pH values, including the means and standard deviations (*n* = 3).
**Figure S2:** Point of zero charge (pHpzc) curve for the *Salvinia molesta* biomass.

## Data Availability

The datasets generated and analyzed in the current study are available from the corresponding author upon reasonable request.
