# Supplementary material for: Valorization of Pretreated Salvinia molesta Biomass for Ciprofloxacin Biosorption: Kinetic Performance, pH‐Dependent Mechanisms, and Circular Economy Implications
Source: Water Environ Res. 2026 Feb 15;98(2):e70304. doi: 10.1002/wer.70304 (PMC12907039; doi:10.1002/wer.70304)
Supplement: Supplementary file 1 — Table S1: Mean ± standard deviation of ciprofloxacin concentrations (μg/L) for each time point and pH condition, with and without biomass. Figure S1: HPLC chromatograms and calibration curves were used for ciprofloxacin quantification, including the detection and quantification limits of the method. Table S2: qt values (μg/g) were calculated across all time points and pH values, including the means and standard deviations (n = 3). Figure S2: Point of zero charge (pHpzc) curve for the Salvinia molesta biomass. [file WER-98-e70304-s001.docx]

**Valorization of Pre-treated *Salvinia molesta* Biomass for Ciprofloxacin Biosorption: Kinetic Performance, pH-Dependent Mechanisms, and Circular Economy Implications**

Leticia Yoshie Kochi^1^, Raizza Zorman Marques^1^, Lia Sumie Nakao^2^, Marcelo Pedrosa Gomes^1*^

^1^Laboratório de Fisiologia de Plantas sob Estresse, Departamento de Botânica, Setor de Ciências Biológicas, Universidade Federal do Paraná, Avenida Coronel Francisco H. dos Santos, 100, Centro Politécnico Jardim das Américas, C.P. 19031, 81531-980, Curitiba, Paraná, Brazil.

^2^Departamento de Patologia Básica, Setor de Ciências Biológicas, Universidade Federal do Paraná, Avenida Coronel Francisco H. dos Santos, 100, Centro Politécnico Jardim das Américas, C.P. 19031, 81531-980, Curitiba, Paraná, Brazil.

*Corresponding author: M. P. Gomes at [marcelo.gomes@ufpr.br](mailto:marcelo.gomes@ufpr.br)

**Supplementary Materials**

**Table S1.** Mean ± standard deviation of ciprofloxacin concentrations (µg/L) for each time point and pH condition, with and without biomass.

| **Without biomass** | | | | |
| --- | --- | --- | --- | --- |
| **Time (min)** | **pH 4.0** | **pH 6.0** | **pH 7.0** | **pH 8.0** |
| 5 | 1.44 ± 0.04 | 1.43 ± 0.03 | 1.43 ± 0.03 | 1.44 ± 0.03 |
| 10 | 1.45 ± 0.03 | 1.45 ± 0.02 | 1.45 ± 0.02 | 1.45 ± 0.03 |
| 20 | 1.45 ± 0.04 | 1.45 ± 0.03 | 1.45 ± 0.03 | 1.45 ± 0.03 |
| 30 | 1.45 ± 0.03 | 1.46 ± 0.02 | 1.45 ± 0.03 | 1.45 ± 0.03 |
| 60 | 1.45 ± 0.04 | 1.46 ± 0.03 | 1.45 ± 0.03 | 1.46 ± 0.02 |
| **With biomass** | | | | |
| **Time (min)** | **pH 4.0** | **pH 6.0** | **pH 7.0** | **pH 8.0** |
| 5 | 0.89 ± 0.06 | 0.56 ± 0.07 | 0.68 ± 0.08 | 0.75 ± 0.05 |
| 10 | 0.73 ± 0.06 | 0.27 ± 0.05 | 0.40 ± 0.06 | 0.47 ± 0.06 |
| 20 | 0.60 ± 0.07 | 0.22 ± 0.04 | 0.36 ± 0.06 | 0.45 ± 0.06 |
| 30 | 0.58 ± 0.04 | 0.21 ± 0.04 | 0.35 ± 0.05 | 0.44 ± 0.06 |
| 60 | 0.57 ± 0.03 | 0.20 ± 0.04 | 0.34 ± 0.05 | 0.43 ± 0.06 |


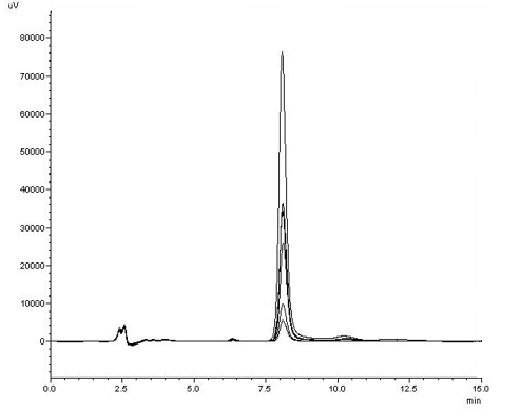


**Figure S1.** HPLC chromatograms and calibration curves were used for ciprofloxacin quantification, including the detection and quantification limits of the method.

**Table S2.** qt values (µg/g) were calculated across all time points and pH values, including the means and standard deviations (n = 3).

| **Time (min)** | **pH 4.0** | **pH 6.0** | **pH 7.0** | **pH 8.0** |
| --- | --- | --- | --- | --- |
| 0 | -0.0 ± 3.3e-05 | 0.0 ± 0.0 | 0.0 ± 4e-06 | 0.0 ± 0.0 |
| 5 | 0.000256 ± 3.7e-05 | 0.000111 ± 3e-06 | 5.6e-05 ± 3.8e-05 | 3.7e-05 ± 4.2e-05 |
| 10 | 0.000246 ± 5e-06 | 0.000233 ± 8.4e-05 | 7.6e-05 ± 1e-05 | 2.2e-05 ± 5.5e-05 |
| 15 | 0.000232 ± 5.7e-05 | 0.000282 ± 0.0 | 0.000244 ± 6e-05 | -3.1e-05 ± 5.5e-05 |
| 30 | 0.000256 ± 7.9e-05 | 0.000282 ± 0.0 | 0.000301 ± 1.9e-05 | 3.7e-05 ± 3.9e-05 |
| 45 | 0.000285 ± 3e-06 | 0.000282 ± 0.0 | 0.000324 ± 4e-06 | -3e-06 ± 4.3e-05 |
| 60 | 0.000276 ± 4e-06 | 0.000282 ± 0.0 | 0.00036 ± 2.8e-05 | -1e-06 ± 1.2e-05 |

**
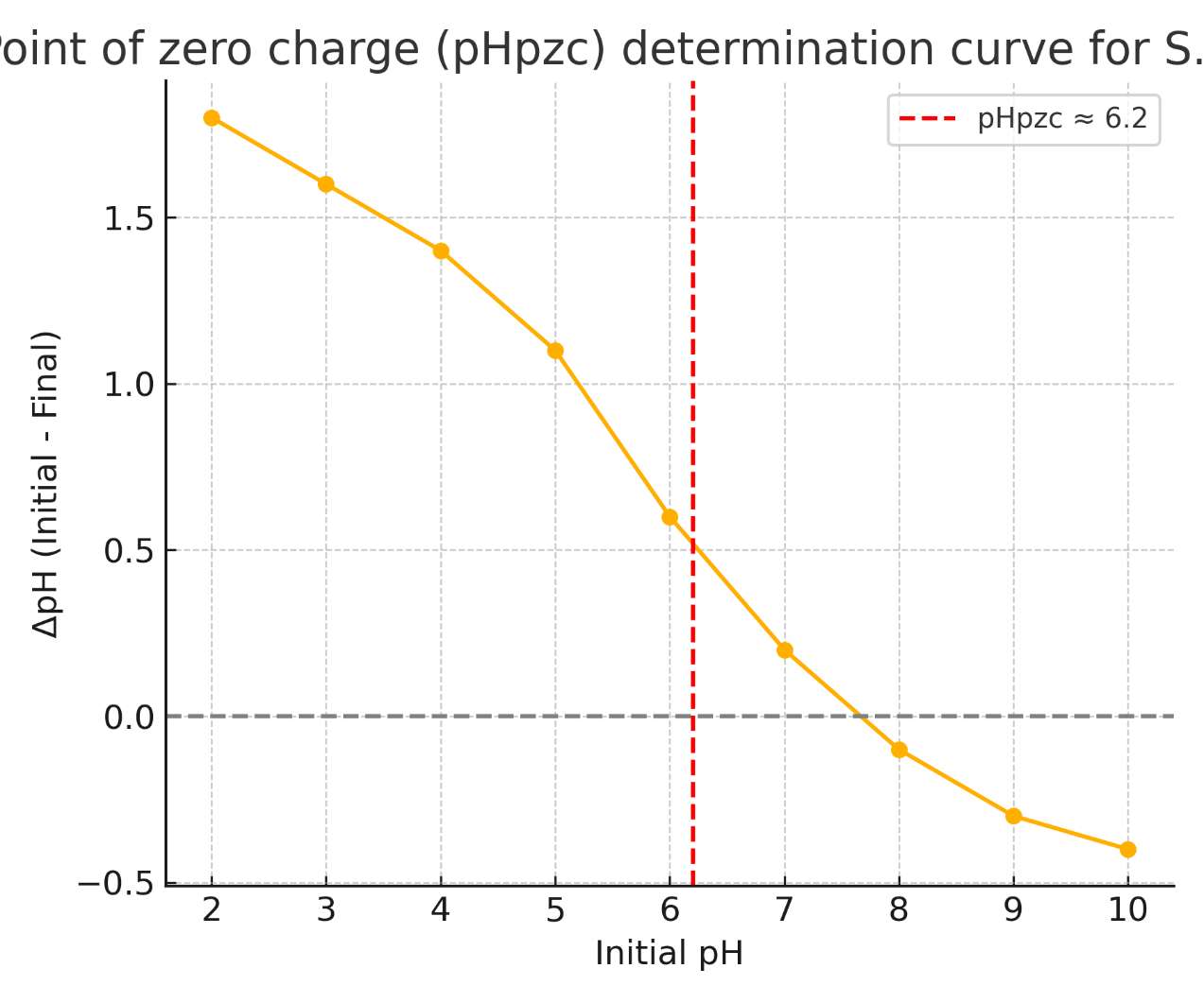
Figure S2.** Point of zero charge (pHpzc) curve for *the S. molesta* biomass.
